# Limited Potential to Reverse Deafness Caused by Mutation of *Myo7a*

**DOI:** 10.1007/s10162-026-01050-2

**Published:** 2026-04-20

**Authors:** Daniel R. Pentland, Jack Blackburn, Lauren Witting, Darcey A. Kirwin, Karen P. Steel

**Affiliations:** 1 Wolfson Sensory, Pain and Regeneration Centre, King’s College London, Guy’s Campus, London SE1 1UL, UK

**Keywords:** Myo7a, Genetics, Mouse, Hearing loss reversal

## Abstract

**Purpose:**

*MYO7A* is involved in several forms of deafness in humans and mice, and in this study we aimed to investigate if the hearing loss could be reversed after its onset.

**Methods:**

A knockdown allele of *Myo7a* in the mouse, *Myo7a*^*tm1a*^, was characterised by recording ABR thresholds at ages from 4 weeks to 6 months old and measuring the amount of hair cell degeneration at 4 weeks old. Scanning electron microscopy was used to assess the condition of stereocilia bundles. A tamoxifen-inducible Flp recombinase was used to activate expression of *Myo7a* in *Myo7a*^*tm1a/tm1a*^ homozygotes at 4 weeks old by excising the transcription disruption cassette in the tm1a allele allowing expression of the *Myo7a* gene, and ABRs were recorded before and after activation of the gene.

**Results:**

*Myo7a*^*tm1a*^ was found to be a recessive allele causing reduced transcription and early onset profound deafness. Some hair cell loss was found at 4 weeks old, and scanning electron microscopy showed *Myo7a*^*tm1a*^ severely affects stereocilia morphology and organisation. Activation of *Myo7a* expression at 4 weeks old results in very small improvements in ABR thresholds of *Myo7a*^*tm1a/tm1a*^ mice at 12 and 18 kHz at 6 and 8 weeks old but there were no responses to sound by 14 weeks old.

**Conclusions:**

It is likely to be challenging to reverse hearing loss due to very early developmental defects of stereocilia organisation.

## Introduction

*Myo7a* encodes an unconventional myosin, myosin-7a, which, in the inner ear, is located in a tripartite complex with SANS and harmonin at the upper tip link density of sensory hair cell stereocilia where it is implicated in tensioning the tip link to optimise the opening probability of the MET channel [1, 2]. Mutations in *MYO7A* cause both nonsyndromic and syndromic deafness in human populations, including the deaf-blind disorder Usher syndrome type 1B (USH1B) [3]. Usher syndrome type 1 is the most severe type of Usher syndrome with type 1B being the most common form, characterised by congenital bilateral sensorineural hearing loss accompanied by vision loss caused by retinitis pigmentosa in early childhood and vestibular dysfunction causing balance difficulties [4, 5]. Autosomal recessive and dominant non-syndromic hearing loss, DFNB2 [6, 7] and DFNA11 [8] respectively, are also caused by *MYO7A* mutations.

As with *MYO7A* mutations in the human population, a range of phenotypes have been identified in previously studied *Myo7a* mouse mutants. The *shaker-1* mouse was first described in the 1920 s [9] but it was not until the 1990 s that the causative mutation was found to be in the *Myo7a* gene [10]. To date, more than 20 different *shaker-1* mutations have been recognised (https://www.informatics.jax.org/allele/summary?markerId=MGI:104510), and although these mutations all result in hearing loss, head-bobbing and circling, they do have differing severities [11]. For instance, the *Myo7a*^*6J*^ and *Myo7a*^*816SB*^ homozygote mutants exhibit severe stereocilia bundle developmental defects, resulting in gross stereocilia disorganisation as early as postnatal day 3 (P3) [11]. However, the original *shaker-1* mutation, *Myo7a*^*sh1*^, has reasonably normal stereocilia bundles with a normal V-shape but fewer stereocilia even at P15 [11]. These differences in stereocilia phenotypes were reflected in the hearing ability of these mice, with *Myo7a*^*6J*^ and *Myo7a*^*816SB*^ homozygotes having more severe hearing loss early in life compared to *Myo7a*^*sh1*^ [11]. Similarly, *Myo7a*^*ewaso*^ mice show profound deafness from early age with concomitant circling behaviour, however, *Myo7a*^*dmbo2*^ mice have severe progressive hearing loss without vestibular dysfunction [12]. A further recently studied *Myo7a* mutant, *Myo7a*^*tm1b*^, also has sensorineural hearing loss with homozygotes displaying profound deafness and heterozygotes having severe hearing loss at mid to high frequencies. This was accompanied by inner and outer hair cell loss as well as spiral ganglion neuron degeneration in both *Myo7a*^+*/tm1b*^ and *Myo7a*^*tm1b/tm1b*^ mice [13]. No *Myo7a* mouse mutant has yet been found to recapitulate the vision defect of USH1B patients [13, 14] possibly due to the differential expression of Myo7a in, and structure of, mouse and primate retinas [13, 15].

There are currently no effective treatments for *MYO7A* mediated deafness, blindness, or vestibular dysfunction, highlighting a crucial need to explore the potential to reverse hearing loss caused by *Myo7a* mutations. Recent gene therapy studies involving *Myo7a* mouse mutants have had some success. A dual-AAV vector-based approach improved vestibular function and cochlear hair cell survival but not auditory function in the *Myo7a*^*4626SB/4626SB*^ mutant [16] and a lentiviral vector strategy improved both auditory and vestibular function in the *shaker-1 Myo7a*^*sh1*^ mutant [17]. However, these studies deliver the *Myo7a* cDNA at an age before hearing impairment has fully manifested, which poses challenges for translation to humans because the equivalent stages occur during gestation in humans. It is, therefore, important to investigate if *Myo7a* mediated hearing loss can be recovered later in life, at an age which would be easier to translate to the clinic.

The purpose of this study was to investigate if an existing hearing loss, caused by *Myo7a* disruption, can be reversed. To this end, a genetic approach was used to activate *Myo7a* expression at four weeks old, after the onset of hearing in the mouse, utilising a ‘knockout-first’ mouse mutant which contains a tm1a transcription disruption cassette [18] between *Myo7a* exons 9 and 10. The presence of this large DNA cassette reduces transcription of *Myo7a* thereby producing a knockdown rather than a knockout mutant. The cassette can be removed by Flp recombinase, reverting the mutation to wildtype. A tamoxifen-inducible optimised Flpe recombinase called Flpo was used [19] to activate expression of *Myo7a* in *Myo7a*^*tm1a/tm1a*^ homozygote mice, as previously used to successfully recover hearing in a *Spns2*^*tm1a*^ mutant [20].

We show that *Myo7a*^*tm1a*^ is a recessive allele causing reduced transcription of the gene and early onset profound deafness. Scanning electron microscopy (SEM) was used to assess stereocilia organisation, showing *Myo7a*^*tm1a*^ severely affects stereocilia morphology and organisation. Activation of *Myo7a* expression at four weeks old results in very small improvements in ABR thresholds of *Myo7a*^*tm1a/tm1a*^ mice at 12 and 18 kHz at six and eight weeks, but this is not stable with age, with no responses to sound stimuli by fourteen weeks old.

## Materials and Methods

### Ethics Statement

Mouse studies were carried out in accordance with UK Home Office regulations and the UK Animals Scientific Procedures Act 1986 (ASPA) under UK Home Office licences. The study was approved by the King’s College London Animal Welfare and Ethical Review Body (AWERB). Mice were maintained in SPF conditions with constant access to food, water and bedding, on a 12 h on/12 h off lighting system, and were culled using methods approved under the licences to minimise any possibility of suffering. Any mice showing signs of ill health (eg abnormal behaviour, coat condition) were culled humanely and not included in the data analysis.

### Mice

**Fig. 1:**
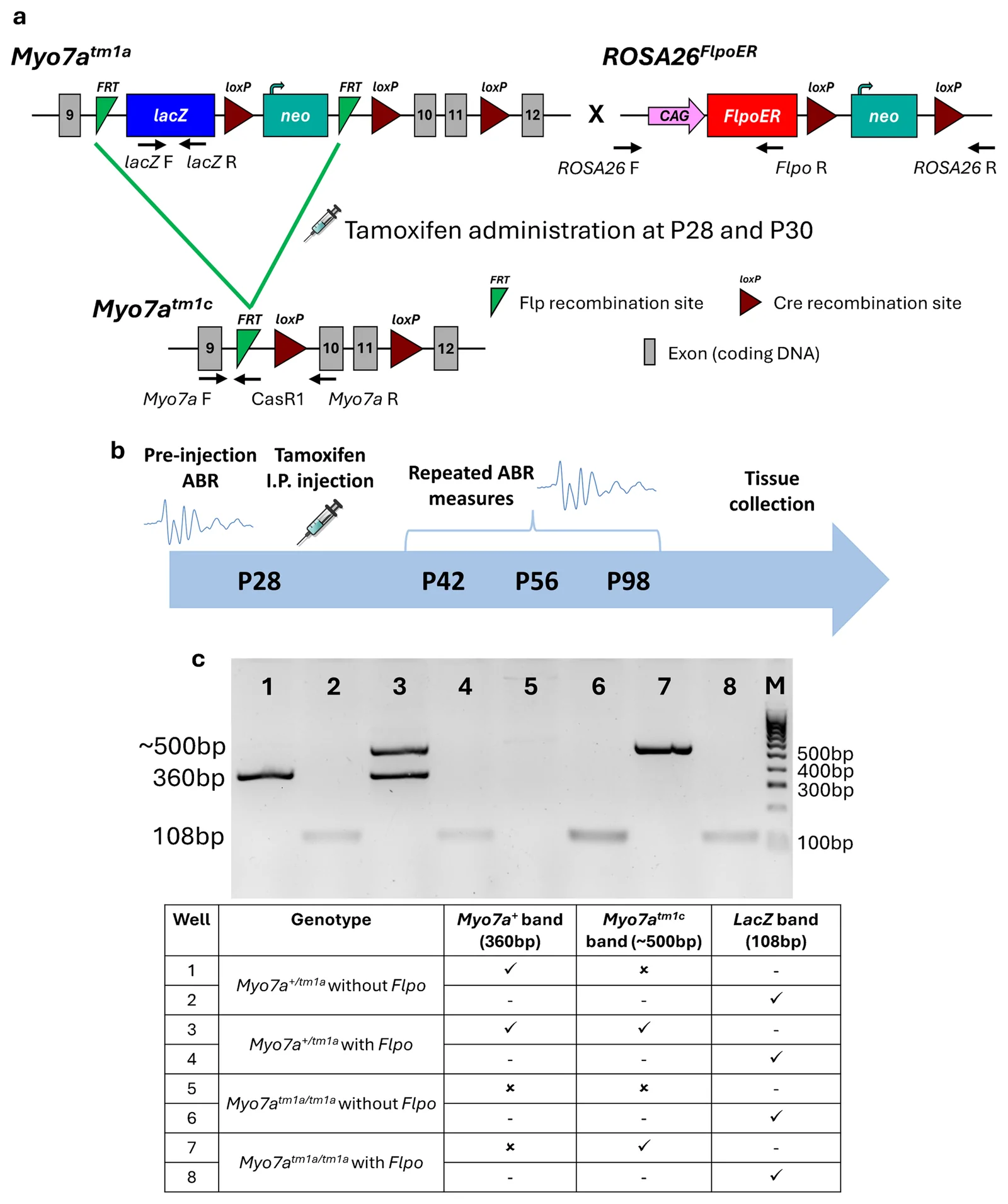
*Myo7a* alleles and experimental design. **a** Diagram detailing the design of the *Myo7a*^*tm1a*^ and *Myo7a*^*tm1c*^ alleles; grey boxes denote exons, green and brown triangles show *FRT* and *loxP* sites respectively, the blue box shows the *lacZ* gene and the teal box shows the neomycin resistance gene. Arrows indicate binding sites of genotyping primers. Upon tamoxifen administration to mice carrying the *ROSA26*^*FlpoER*^, the Flpo recombinase mediates recombination between *FRT* sites, removing the transcription disruption cassette and creating the *Myo7a*^*tm1c*^ allele which is functional. **b** Schematic showing the experimental timeline of this study; the mice received tamoxifen at P28 and P30, after a pre-tamoxifen baseline ABR at four weeks old (P28), and follow-up ABRs were performed at six, eight and fourteen weeks old prior to inner ear collection. All mice received tamoxifen regardless of genotype. **c** Agarose gel showing the genomic PCR product using pinna tissue, showing the excision of the tm1a cassette: *Myo7a* F and *Myo7a* R primers (panel a) produced either a 360 bp product from the wildtype *Myo7a*^+^ allele or a ~ 500 bp product from the *Myo7a*^*tm1c*^ allele if the tm1a cassette has been excised. Thus, genomic template from *Myo7a*^*tm1a/tm1a*^ with *Flpo* mice produced a single ~ 500 bp band, and template from *Myo7a*^*tm1a/tm1a*^ without *Flpo* mice did not produce a band. If a control *Myo7a*^+*/tm1a*^ mouse carried *Flpo*, 2 bands of 360 bp and ~ 500 bp were produced. If any unexcised tm1a cassette remains, the *lacZ* F and *lacZ* R primers (panel a) produced a 108 bp product from the *Myo7a*^*tm1a*^ allele; this reaction suggested some uncut *Myo7a*^*tm1a*^ remained in the samples from *Myo7a*^*tm1a/tm1a*^ with *Flpo* mice consistent with incomplete expression of Myo7a in hair cells

The *Myo7a*^*tm1a(EUCOMM)Wtsi*^ mutant mice (abbreviated to *Myo7a*^*tm1a*^) were generated on a C57BL/6N genetic background at the Wellcome Sanger Institute as part of the Mouse Genetics Project [21]. The *Myo7a*^*tm1a*^ allele is a ‘knockout-first’ design with a large transcription-disruption cassette between exons 9 and 10 designed to knockdown gene expression (Fig. 1a) [18]. For the initial *Myo7a*^*tm1a*^ characterisation experiments, *Myo7a*^*tm1a/tm1a*^ homozygotes and *Myo7a*^+*/tm1a*^ heterozygotes were used alongside *Myo7a*^+*/*+^ littermate controls. The *Cdh23*^*ahl*^ variant, which is known to cause early onset high frequency hearing loss [22, 23] is present in the C57BL/6N genetic background so we crossed the *Myo7a*^*tm1a/tm1a*^ mutation to a targeted repaired allele of *Cdh23*^*ahl*^, *Cdh23*^*ahl*+*em3H*^, to avoid any confounding effects.

To determine the effect of postnatally activating *Myo7a* expression on auditory phenotypes, the *Myo7a*^*tm1c*^ allele was generated by crossing *Myo7a*^*tm1a*^ mutants with B6N.129S6(Cg)-*Gt(ROSA)26Sor*^*tm3(CAG−flpo/ERT2)Alj*^/J (abbreviated to *Flpo*) mice ubiquitously expressing a tamoxifeninducible optimised Flpe recombinase called *Flpo* [19]. The large inserted cassette of the *Myo7a*^*tm1a*^ allele was excised by Flpo recombinase-mediated recombination between *FRT* sites following administration of tamoxifen, restoring normal transcription of *Myo7a* (Fig. 1a). Mice carrying the *Myo7a*^*tm1a*^ and *Flpo* alleles were bred to produce littermates with the following genotypes: *Myo7a*^+*/*+^ and *Myo7a*^+*/tm1a*^ with or without *Flpo* (controls), *Myo7a*^*tm1a/tm1a*^ homozygotes without *Flpo*, and *Myo7a*^*tm1a/tm1a*^ homozygotes with *Flpo*, and all mice in these litters were exposed to tamoxifen at either P4 or P28 as described below to activate Flpo. Where possible, experimental mice were sex and age-matched to control animals. Both male and female mice were included in the study but no difference in auditory phenotype was observed between them.

### Data Availability

Both mouse alleles used are available from public archives. Unprocessed data will be provided on request if not included in the manuscript.

### Genotyping

Genomic DNA was extracted from pinna skin tissue and used as a template for short-range PCRs using primers detailed in Table 1 (primer binding locations indicated in Fig. 1a). If the tm1a cassette is present at the *Myo7a* locus, the *Myo7a* wildtype reaction will not be successful as the primer binding sites are too far apart. Confirmation of the presence of the tm1a cassette was established with a second reaction using primers designed to bind in the *lacZ* gene within the cassette. This reaction is only successful if the tm1a cassette is present, however, it does not distinguish between heterozygote or homozygote mice. Mice with a *Myo7a* wildtype band (360 bp) but no *Myo7a*^*tm1a*^ band (178 bp) or *lacZ* band (108 bp) are considered *Myo7a*^+*/*+^, mice with a *Myo7a* wildtype band (360 bp) and both *Myo7a*^*tm1a*^ and *lacZ* bands are considered *Myo7a*^+*/tm1a*^, and mice without a *Myo7a* wildtype band but have both *Myo7a*^*tm1a*^ and *lacZ* bands are considered *Myo7a*^*tm1a/tm1a*^. When required, the presence/absence of *Flpo* was also determined via shortrange PCR of pinna tissue using primers detailed in Table 1. If the *Flpo* gene is not present at the *ROSA26* locus, the *ROSA26* wildtype reaction will produce a product of 603 bp, whereas if the *Flpo* gene is present, the *ROSA26 Flpo* reaction a PCR product of 309 bp will be made.

To determine if the tm1a cassette has been excised from *Myo7a*^*tm1a/tm1a*^ with *Flpo* mice, a further short-range PCR was performed on pinna tissue from mice which had received tamoxifen at either four weeks old or P4. If *Flpo*-mediated recombination has been successful, the *Myo7a* wildtype reaction will now produce a band of ~ 500 bp instead of failing. This is larger than the usual *Myo7a* wildtype band due to the continued presence of the *FRT* and *loxP* sites (Fig. 1a and c). The *lacZ* reaction was also run again to determine if there is any unexcised tm1a cassette remaining (Fig. 1c).

### Digital Droplet PCR (ddPCR)

To assess the amount of knockdown of gene expression, whole inner ears of four week old mice were dissected from the temporal bone in RNALater (AM7024, ThermoFisher) and snap frozen in liquid nitrogen. Total RNA was extracted from one inner ear per mouse using a Lexogen SplitRNA kit (SKU008.48, Lexogen). A Nanodrop spectrophotometer was used to determine RNA concentration and purity. The RNA for all samples was normalised to the same concentration and cDNA was synthesised using a Superscript IV VILO kit (11,766,500, Invitrogen) according to manufacturer’s instructions. Digital droplet PCR (ddPCR) was used with Taqman™ probes to determine the quantity of *Myo7a* (Taqman™ probe ID: Mm00485372_m1, ThermoFisher) mRNA relative to the house keeping gene *Hprt1* (Taqman™ probe ID: Mm00446968_m1, ThermoFisher). The Taqman™ probe for *Myo7a* was selected to bind downstream of the tm1a cassette. All equipment and consumables were selected to be compatible with the Bio-Rad QX100™ Digital Droplet PCR system and carried out as per manufacturer’s instructions.

### Auditory Brainstem Response (ABR) Recordings

ABRs were recorded in mutant mice and littermate controls at a range of ages from four to twenty-six weeks old, to assess auditory function. The mice were anaesthetised by intraperitoneal injection with 0.1 mg/g Ketamine (Ketamidor, Chanelle Pharma) and 0.01 mg/g Xylazine (Rompun, Bayer Animal Health). Mice were kept warm on a heated mat during recovery, and recovery was promoted using intraperitoneal injection of 1 mg/kg Atipamezole (Antisedan, Pfizer). Auditory-evoked brainstem potentials were measured as previously described [24]. Briefly, mice were placed on a 37 °C heated blanket inside a sound-attenuating booth and subcutaneous recording needle electrodes were inserted in the skin on the vertex (active), left bullae (reference) and right bullae (ground). Auditory stimuli (10 µs duration broadband click, and 5 ms duration tone pips at frequencies between 3–42 kHz) were presented from 0–95 dB SPL in 5 dB steps as free-field sounds from a loudspeaker positioned 20 cm in front of the mouse’s interaural axis. The evoked responses were digitised, amplified and bandpassfiltered between 300–3000 Hz, and 256 responses were averaged for each frequency and intensity combination to generate a waveform. ABR thresholds at each frequency were determined according to the lowest intensity level at which a feature of the ABR waveform can be visually detected.

### Tamoxifen-induced Gene Recombination

For the four week old mouse cohort (Fig. 1b), tamoxifen (20 µg/µl) dissolved in corn oil (C8267, Sigma-Aldrich) was injected intraperitoneally at a dose of 0.2 mg/g body weight. Each mouse received 2 doses separated by 48 h (at P28 and P30). ABRs were recorded the same day prior to the first tamoxifen injection to get a ‘baseline’ ABR. Follow-up ABRs were then performed at six, eight and fourteen weeks of age to track hearing over time in the same animals. For the P4 mouse cohort (Supplementary Fig. 1a), tamoxifen was injected intraperitoneally at 0.2 mg/g body weight to the lactating mothers when their litters were P4. ABRs were performed at four, six and eight weeks of age; ‘baseline’ ABRs prior to tamoxifen administration was not possible for the P4 cohort as hearing does not develop until approximately P12 in mice. Entire litters, regardless of genotype, received tamoxifen.

### Immunolabelling and Confocal Imaging

For hair cell quantification in *Myo7a*^*tm1a/tm1a*^ mice, the inner ears of P28 mice (*Myo7a*^*tm1a/tm1a*^ with littermate controls) were dissected away from the temporal bone and fixed in 4% PFA in phosphate-buffered saline (PBS) for 3 h and decalcified overnight in 0.1 M EDTA. The organ of Corti was dissected out and permeabilised with 5% Tween-20 in PBS for 30 min before incubation for 2 h in blocking solution (10% normal horse serum with 0.3% Triton X-100 in PBS). After blocking, samples were incubated overnight at 4 °C with goat anti-calretinin (1:400, CG1, Swant) and goat antiprestin (1:100, C-16, Santa-Cruz Biotechnology) primary antibodies in antibody solution (5% normal horse serum with 0.15% Triton X-100 in PBS), to label inner and outer hair cells respectively. Samples were then washed with PBS before incubation with Alexa Fluor 488-conjugated antigoat secondary antibody (1:300, A21082, Invitrogen) in antibody solution for 1 h. The stained organ of Corti pieces were washed with PBS before being mounted using Prolong Gold mounting media with DAPI (P36931, Life Technologies). All the above steps were carried out at room temperature. Samples were imaged using a Zeiss LSM710 confocal microscope interfaced with ZEN Black software (version 14.0.17.201) using 405 nm and 488 nm lasers with a 40 × 1.3 NA oil immersion plan-apo objective lens. Z-stacks were taken with a step size of 1 µm and maximum intensity projections created for visualisation and analysis. The best frequency regions were determined using the ‘Measure Line’ frequency mapper ImageJ plugin from the Eaton-Peabody Laboratories Histology Core website (https://masseyeandear.org/research/otolaryngology/eaton-peabody-laboratories/histology-core). Hair cell quantification of a 200 µm length of the organ of Corti per frequency region was performed using the cell counter plugin in ImageJ. Hair cell counts were then normalised to cells per 100 µm.

For quantification of Flpo-mediated recombination and subsequent Myo7a expression after tamoxifen administration, cochleae of P105 mice (*Myo7a*^*tm1a/tm1a*^ with *Flpo, Myo7a*^*tm1a/tm1a*^ without *Flpo*, and littermate controls) were collected one week after their P98 ABRs (Fig. 1b). Samples were collected as described above, except the 0.1 M EDTA decalcification step was extended to 72 h. After dissection, organ of Corti pieces were stained as above with the following primary antibodies: goat anti-calretinin (1:400, CG1, Swant), goat anti-prestin (1:100, C-16, Santa-Cruz Biotechnology) and rabbit anti-Myo7a (1:300, 25–6790, Proteus). Following primary antibody incubation, samples were washed with PBS before incubation with the secondary antibodies Alexa Fluor 488-conjugated anti-rabbit (1:300, A21206, Molecular Probes) and Alexa Fluor 633-conjugated anti-goat (1:300, A21082, Invitrogen). Stained samples were imaged using a Zeiss LSM710 confocal microscope interfaced with ZEN Black software (version 14.0.17.201) using 405 nm, 488 nm, and 633 nm lasers with a 20 × 0.8 NA plan-apo air objective lens. Tile scan images were taken (10% overlap, 0.7 threshold) with a 1 µm Z-interval to image the entirety of the organ of Corti pieces. For quantification, the full organ of Corti length was divided into 5% sections from the apex to the base using the ‘Measure Line’ ImageJ plugin detailed above, the total number of inner and outer hair cells, as well as the number of these expressing Myo7a, were counted using the cell counter ImageJ plugin.

### Scanning Electron Microscopy (SEM)

The cochleae of P63 mice which had received tamoxifen at P4 were dissected away from the temporal bone and fixed in 2.5% glutaraldehyde (AA012, Bangs Laboratories) in 0.1 M sodium cacodylate buffer (ARG1500, Agar Scientific) with 3 mM calcium chloride for 5 h at room temperature followed by overnight incubation in the fixative at 4 °C. Cochleae were subsequently washed and dissected in PBS to remove the encasing bone and expose the underlying sensory tissue. The dissected cochleae were then processed for SEM using the previously published osmiumthiocarbohydrazide (OTOTO) method [25]. The samples were dehydrated through an ascending ethanol series from 20 to 100%, critical point dried using a CPD300 (Leica), mounted on stubs with conductive silver paint (123–9911, RS Pro) and imaged using the secondary electron detector on a JEOL JSM 7800 F Prime SEM in room temperature mode. Images were taken using 5 kV accelerating voltage. Inner ears from at least 3 control, 3 *Myo7a*^*tm1a/tm1a*^ with *Flpo*, and 3 *Myo7a*^*tm1a/tm1a*^ without *Flpo* mice were examined.

## Statistical Analyses

All statistical analyses were performed using GraphPad Prism (version 10.6.1). The distribution of all datasets was tested for normality using a Shapiro–Wilk test. For datasets with normal distribution, statistical significance was determined using two-way ANOVAs followed by Tukey or Šidák multiple comparisons tests. For datasets which did not pass the normal distribution test, principally the ABR datasets, statistical significance was established using Kruskal–Wallis nonparametric tests followed by Dunn’s multiple comparisons tests. All numerical data is presented as mean ± 1 standard deviation (SD). In all experiments, the unit used for statistical comparison is the mouse. Littermates were used as controls on the same day as the mutants, in random order.

## Results

### *Myo7a*^*tm1a/tm1a*^ Homozygotes have Significantly Reduced *Myo7a* Expression

**Fig. 2:**
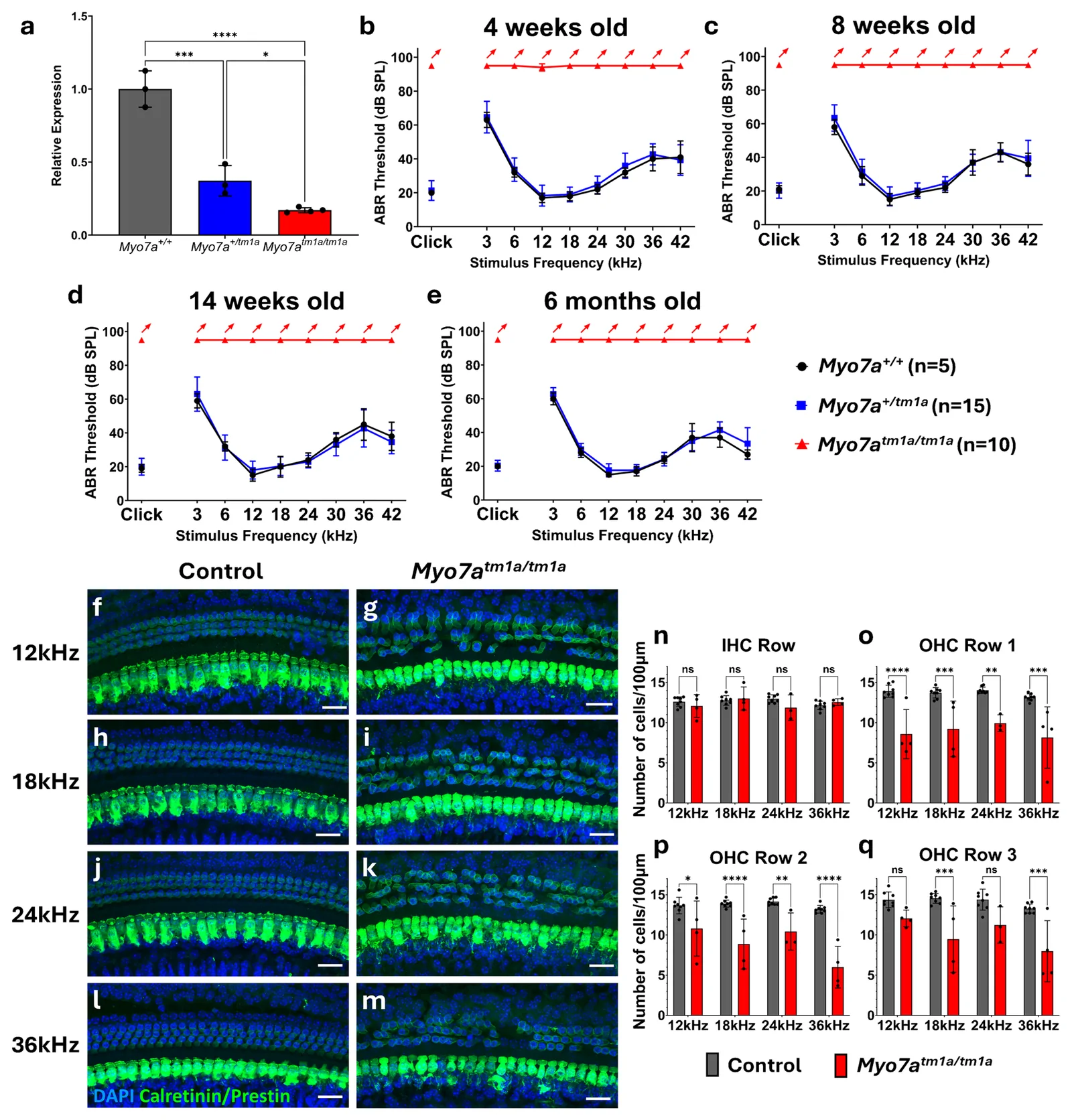
The *Myo7a*^*tm1a*^ allele is recessive and causes profound hearing loss. **a** Relative inner ear mRNA expression level at four weeks old of *Myo7a* in *Myo7a*^+*/*+^ (*n* = 3), *Myo7a*^+*/tm1a*^ (*n* = 3), *Myo7a*^*tm1a/tm1a*^ (*n* = 4). One-way ANOVA with Tukey’s multiple comparison test: *p < 0.05, ***p < 0.001, ****p < 0.0001. **b**-**e** ABR thresholds of *Myo7a*^+*/*+^ (black), *Myo7a*^+*/tm1a*^ (blue), and *Myo7a*^*tm1a/tm1a*^ (red) mice at four, eight, fourteen weeks and 6 months old between 3–42 kHz. *Myo7a*^*tm1a/tm1a*^ mice displayed profound hearing loss from four weeks old across all frequencies tested. Data points indicate the lowest sound level (mean ± 1 SD) at which features of the ABR waveform start to appear. Data points at 95 dB SPL indicate no response up to the highest sound level used. Responses of *Myo7a*^*tm1a/tm1a*^ mice were significantly raised compared to both *Myo7a*^+*/tm1a*^ and *Myo7a*^+*/*+^ mice at all ages (*p* < 0.01, Kruskal–Wallis with Dunn’s multiple comparison test). **f**-**m** Confocal images of organ of Corti whole mounts from control and *Myo7a*^*tm1a/tm1a*^ mice at four weeks old. IHCs and OHCs labelled with anti-calretinin and anti-prestin respectively (green), nuclei labelled with DAPI (blue). Scale bars = 20 µm. **n**-**q** Quantification of HCs in control (black, *Myo7a*^+*/*+^ and *Myo7a*^+*/tm1a*^) and *Myo7a*^*tm1a/tm1a*^ (red) mice at 12 kHz, 18 kHz, 24 kHz and 36 kHz best-frequency regions. Two-way ANOVA with Tukey’s multiple comparison test: *p < 0.05, **p < 0.01, ***p < 0.001, ****p < 0.0001, ns = not significant

The *Myo7a*^*tm1a*^ allele contains a large cassette of DNA in the intron immediately upstream of the critical exons 10 and 11, which is predicted to disrupt transcription of *Myo7a* [18]. The degree of knockdown of *Myo7a* mRNA expression was investigated using digital droplet PCR (ddPCR). *Myo7a* expression was significantly reduced to 17 ± 2% in the inner ear of *Myo7a*^*tm1a/tm1a*^ mice compared to wildtype controls (*p* < 0.0001, Tukey multiple comparison test) (Fig. 2a). *Myo7a*^+*/tm1a*^ mice also exhibited a significant reduction down to 37 ± 10% in *Myo7a* inner ear expression compared to wildtype controls (*p* = 0.0001, Tukey multiple comparison test). Interestingly, despite this reduction, no hearing impairment was found in *Myo7a*^+*/tm1a*^ mice (see below).

### *Myo7a*^*tm1a/tm1a*^ Homozygotes are Profoundly Deaf from as Early as Four Weeks Old

*Myo7a* is expressed in inner and outer hair cells and mutations in this gene are widely associated with deafness in both humans and mice. Auditory brainstem response (ABR) measurements were performed at four, eight, fourteen and twenty-six weeks old to track the hearing of *Myo7a*^+*/tm1a*^ and *Myo7a*^*tm1a/tm1a*^ mice compared to wildtype littermate controls. *Myo7a*^*tm1a/tm1a*^ homozygote mice showed no responses up to 95 dB SPL, the maximum stimulus intensity used, across all frequencies tested from as early as four weeks old (Fig. 2b-e). There was no significant difference between ABR thresholds of *Myo7a*^+*/*+^ and *Myo7a*^+*/tm1a*^ mice (*p* > 0.9999, Dunn’s multiple comparison test) all the way up to 6 months of age (Fig. 2e), suggesting the *Myo7a*^*tm1a*^ allele is recessive for this phenotype, despite having reduced levels of transcript (Fig. 2a).

### *Myo7a*^*tm1a/tm1a*^ Homozygotes Have Significant Outer Hair Cell Loss but no Inner Hair Cell Loss at Four Weeks Old

To ascertain if the profound hearing loss in *Myo7a*^*tm1a/tm1a*^ homozygote mice is correlated with sensory hair cell loss, IHCs and OHCs were quantified via confocal microscopy at P28 (Fig. 2f-m). *Myo7a*^*tm1a/tm1a*^ mutants exhibited similar numbers of inner hair cells per 100 µm as littermate controls at 12 kHz (*p* = 0.7660, Šidák multiple comparison test), 18 kHz (*p* = 0.9833, Šidák multiple comparison test), 24 kHz (*p* = 0.1991, Šidák multiple comparison test), and 36 kHz (*p* = 0.8964, Šidák multiple comparison test) best frequency regions (Fig. 2n). However, outer hair cell numbers were significantly reduced across all 3 rows (Fig. 2o-q). As all inner hair cells, and a large proportion of outer hair cells, were still surviving at P28 which is an age when *Myo7a*^*tm1a/tm1a*^ mice are profoundly deaf, this indicates that hair cell loss is not the primary cause of the hearing loss.

### Activation of *Myo7a* Expression at Four Weeks Old Causes a Small Improvement in ABR Thresholds at 12 kHz

**Fig. 3:**
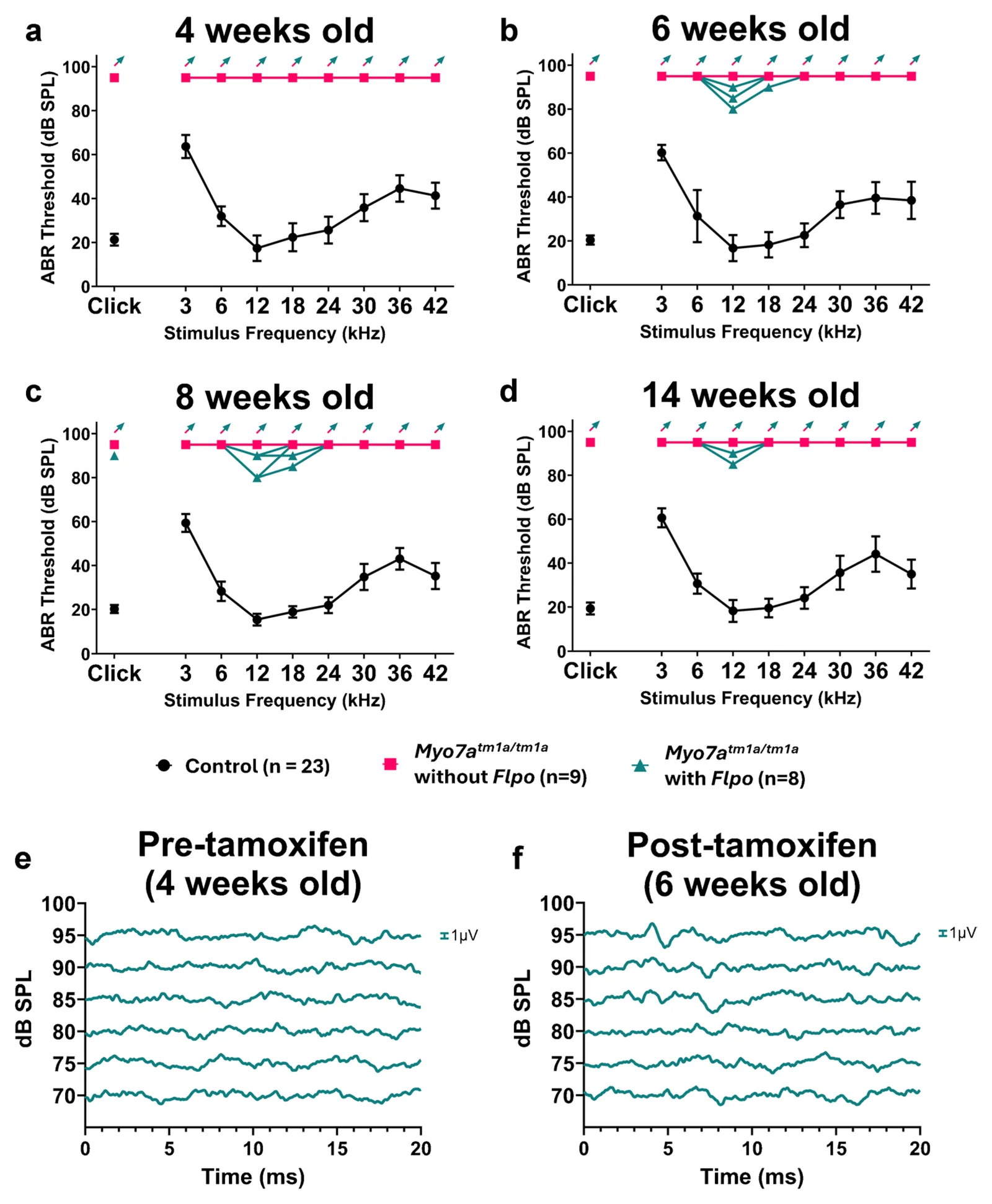
Activation of *Myo7a* expression at four weeks old results in an auditory brainstem response at 12 kHz in *Myo7a*^*tm1a/tm1a*^ mice. **a**-**d** ABR thresholds of control (black – mean ± 1 SD), *Myo7a*^*tm1a/tm1a*^ without *Flpo* (pink – mean ± 1 SD), and *Myo7a*^*tm1a/tm1a*^ with *Flpo* (teal – individual values) mice at four weeks (prior to tamoxifen (TMX) injection), six, eight and fourteen weeks old between 3–42 kHz. Four out of eight *Myo7a*^*tm1a/tm1a*^ with *Flpo* mice produced an ABR at 12 and/or 18 kHz at high decibel levels post tamoxifen administration. Data points indicate the lowest sound level at which features of the ABR waveform start to appear. Data points at 95 dB SPL indicate no response up to the highest sound level used. Responses of both *Myo7a*^*tm1a/tm1a*^ with and without *Flpo* mice were significantly raised compared to control mice (*p* < 0.01, Kruskal–Wallis with Dunn’s multiple comparison test). There was no significant difference between responses of *Myo7a*^*tm1a/tm1a*^ with and without *Flpo* mice. **e**–**f** ABR stacks at 70-95db SPL from a *Myo7a*^*tm1a/tm1a*^ with *Flpo* mouse at 4 weeks old (pre-tamoxifen) and again from the same mouse at 6 weeks old (2 weeks after tamoxifen). The mouse progresses from having no response at 4 weeks old to producing evoked responses to high sound levels at 6 weeks old

We used a genetic approach to determine if the profound deafness exhibited by *Myo7a*^*tm1a/tm1a*^ mutant mice could be reversed by the activation of *Myo7a* gene expression at an age after the onset of hearing loss (Fig. 1a). To this end, we crossed *Myo7a*^*tm1a*^ mice with a strain expressing a tamoxifen-inducible, optimised FLPe recombinase called *Flpo*, to generate *Myo7a*^*tm1a/tm1a*^ mice with or without *Flpo* alongside littermate controls. Tamoxifen was administered to all mice in each litter irrespective of genotype, after a baseline ABR, at P28, and again at P30, to convert the *Myo7a*^*tm1a*^ allele (*Myo7a* expression knockdown) to the *Myo7a*^*tm1c*^ allele (*Myo7a* expression activated) (Fig. 1a). This approach has previously been successful in reversing hearing loss in a *Spns2*^*tm1a*^ mouse mutant [20]. By comparing pre- and post-tamoxifen ABR thresholds in the same mice, we observed that tamoxifen administration at four weeks old led to some responses being detected at very high stimulus levels in 4 out of 8 *Myo7a*^*tm1a/tm1a*^ with *Flpo* mice at 12 kHz (Fig. 3a-d). This is particularly apparent at six weeks old (Fig. 3b) and by eight weeks there is also a small response at 18 kHz as well as 12 kHz (Fig. 3c). Although the improvements in ABR threshold were subtle, it is noteworthy because individual mice progressed from not producing an ABR at all, to being able to detect sound for the first time albeit at high decibel levels (Fig. 3e and f). By fourteen weeks old, the responses of *Myo7a*^*tm1a/tm1a*^ with *Flpo* mice had mostly disappeared (Fig. 3d). As expected, we observed no responses in *Myo7a*^*tm1a/tm1a*^ mice without *Flpo* (Fig. 3a-d), indicating the responses of *Myo7a*^*tm1a/tm1a*^ with *Flpo* mice were indeed due to the tamoxifen-inducible *Flpo* activating *Myo7a* expression.

**Fig. 4:**
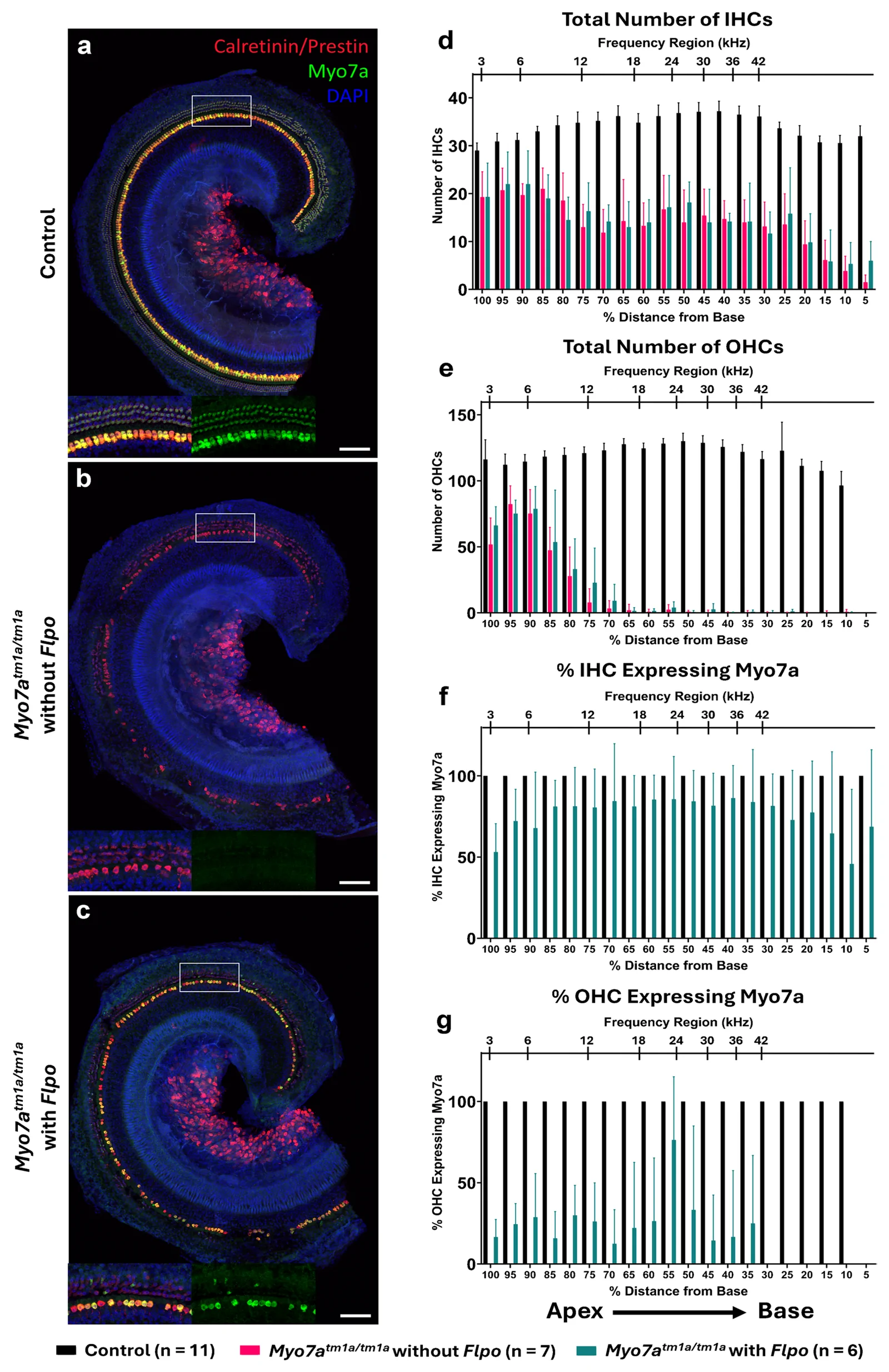
Tamoxifen injections at four weeks old cause activation of Myo7a expression in *Myo7a*^*tm1a/tm1a*^ with *Flpo* mice but not in *Myo7a*^*tm1a/tm1a*^ without *Flpo* mice. **a**-**c** Confocal images of apical pieces of organ of Corti whole mounts from control, *Myo7a*^*tm1a/tm1a*^ without *Flpo*, and *Myo7a*^*tm1a/tm1a*^ with *Flpo* mice at fifteen weeks old. Littermate controls were *Myo7a*^+*/*+^ or *Myo7a*^+*/tm1a*^ either with or without Flpo. Tamoxifen was injected to all mice at both P28 and P30 regardless of genotype. IHCs and OHCs labelled with anti-myoVIIA (green), anti-calretinin (IHCs—red), anti-prestin (OHCs – red), nuclei labelled with DAPI (blue). Scale bars = 100 µm. Regions outlined in white are shown enlarged at the bottom of each panel, with merged channels on the left and the Myo7a channel alone on the right. **d**-**e** Total number of surviving IHCs and OHCs across the entire organ of Corti length. Surviving IHCs and OHCs were significantly reduced in both *Myo7a*^*tm1a/tm1a*^ with and without *Flpo* mice compared to littermate controls (*p* < 0.0001). There was no significant difference in IHC and OHC numbers between *Myo7a*^*tm1a/tm1a*^ with and without *Flpo* mice. **f**-**g** Percentage of surviving IHCs and OHCs which express Myo7a. Across the whole organ of Corti length, the percentage of IHCs expressing Myo7a in *Myo7a*^*tm1a/tm1a*^ with *Flpo* mice was significantly different to both control and *Myo7a*^*tm1a/tm1a*^ without *Flpo* mice (*p* = 0.0005). The percentage of OHCs expressing Myo7a in *Myo7a*^*tm1a/tm1a*^ with *Flpo* mice was also significantly different to both control (*p* < 0.0001) and *Myo7a*^*tm1a/tm1a*^ without *Flpo* mice (*p* = 0.0181). All statistical analyses were performed using Kruskal– Wallis with Dunn’s multiple comparison tests. No OHC values are presented for the basal 5% due to the difficulty in accurately dissecting and counting this region

After completion of the ABRs, to validate if tamoxifen administration at four weeks old results in detectable Myo7a expression, we used immunolabelling of Myo7a in organ of Corti wholemounts from mice at fifteen weeks old. As expected, control mice exhibited Myo7a immunofluorescence in both IHCs and OHCs across the entire length of the organ of Corti (Fig. 4a, f and g), whereas *Myo7a*^*tm1a/tm1a*^ without *Flpo* mice displayed no Myo7a immunofluorescence in either IHCs or OHCs (Fig. 4b, f and g). *Myo7a*^*tm1a/tm1a*^ mice with *Flpo* exhibited Myo7a immunofluorescence in the majority of surviving IHCs across the entire organ of Corti length (Fig. 4c and f), demonstrating that the tamoxifeninducible Flpo recombinase has removed the transcription disruption cassette from the *Myo7a*^*tm1a*^ allele and activated *Myo7a* expression. However, there was a substantially lower proportion of surviving OHCs expressing Myo7a (Fig. 4g). There was significant inner and outer hair cell loss throughout the cochlear duct in both *Myo7a*^*tm1a/tm1a*^ with *Flpo* and *Myo7a*^*tm1a/tm1a*^ without *Flpo* mice, with the greatest degree of degeneration occurring towards the base (Fig. 4d and e). Indeed, there were almost no surviving OHCs in *Myo7a*^*tm1a/tm1a*^ either with or without *Flpo* in the basal 75% of the cochlear duct (Fig. 4e). The mice received tamoxifen at an age (four weeks) when all inner hair cells and large proportions of outer hair cells are still present (Fig. 2f-m), implying that despite activating Myo7a expression, the hair cells are still degenerating, and this could explain why the 12 kHz ABR threshold improvement is not stable as the mice age. Consistent with this, there is no significant difference in overall numbers of surviving IHCs (*p* > 0.9999, Dunn’s multiple comparison test) or OHCs (*p* > 0.9999, Dunn’s multiple comparison test) between *Myo7a*^*tm1a/tm1a*^ with *Flpo* and *Myo7a*^*tm1a/tm1a*^ without *Flpo* mice (Fig. 4d and e). Moreover, although the majority of surviving IHCs in *Myo7a*^*tm1a/tm1a*^ with *Flpo* mice do express Myo7a after tamoxifen administration (Fig. 4f), there is still a significant difference in the percentage of IHCs expressing Myo7a compared to control mice (*p* = 0.0005, Dunn’s multiple comparison test).

### Tamoxifen Administration at P4 Does not Activate *Myo7a* Expression

As tamoxifen administration at younger ages has previously been shown to be more effective at reversing the hearing impairment compared to older ages in the *Spns2*^*tm1a*^ mouse mutant [20], we decided to investigate if the improvement in hearing in *Myo7a*^*tm1a/tm1a*^ with *Flpo* mice would be greater if tamoxifen was administered at P4. Mice at P4 received tamoxifen via the milk of their lactating mothers before undergoing ABRs later (Supplementary Fig. 1a). However, there was very little or no response to sound in *Myo7a*^*tm1a/tm1a*^ mice with and without *Flpo* at four, six and eight weeks old after receiving tamoxifen from P4 (Supplementary Fig. 1b-d). Myo7a immunolabelling was used to assess whether the gene was expressed in IHCs and OHCs of *Myo7a*^*tm1a/tm1a*^ with *Flpo* mice at nine weeks old following early tamoxifen administration at P4. Despite evidence that Flpo recombinase cut the tm1a cassette from *Myo7a*^*tm1a*^ as determined via genomic PCR using pinna tissue (Supplementary Fig. 2a), we observed a lack of expression of Myo7a in *Myo7a*^*tm1a/tm1a*^ with *Flpo* (Supplementary Fig. 2d) which can explain the absence of any rescue of hearing loss.

### *Myo7a*^*tm1a/tm1a*^ Mice Have Severely Disorganised Inner and Outer Hair Cell Stereocilia

**Fig. 5:**
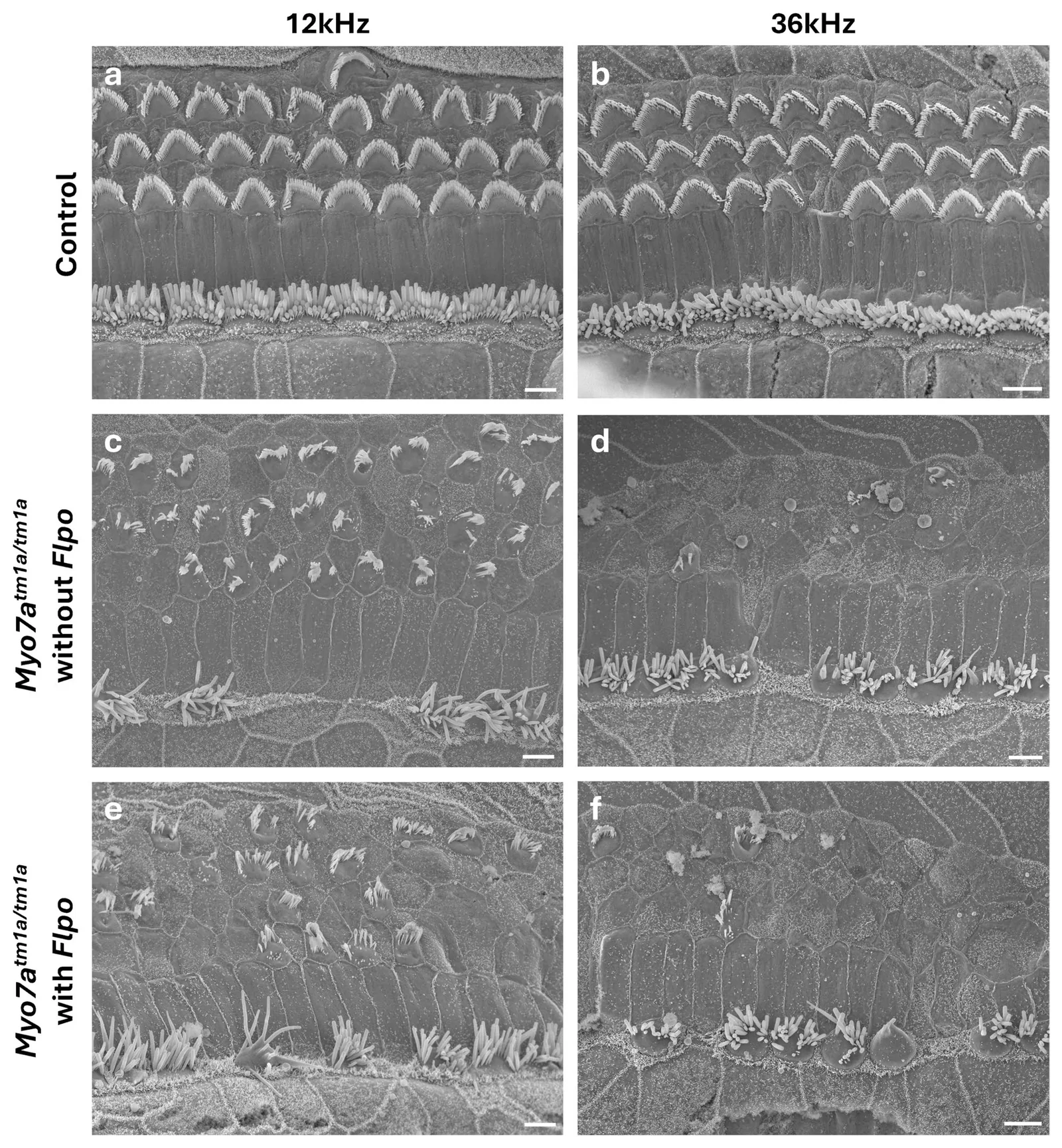
*Myo7a*^*tm1a/tm1a*^ mice have substantial inner and outer hair cell loss along with severely disorganised stereocilia Scanning electron microscopy images of 12 kHz and 36 kHz organ of Corti frequency regions from nine week old mice which had received tamoxifen at P4 via the mother; **a** and **b** control mice (*Myo7a*^+*/tm1a*^ with or without Flpo), **c** and **d** *Myo7a*^*tm1a/tm1a*^ without *Flpo*, **e** and **f** *Myo7a*^*tm1a/tm1a*^ with *Flpo*. All mice received tamoxifen regardless of genotype. Scale bars = 5 µm. *N* = 3 for each genotype

**Fig. 6:**
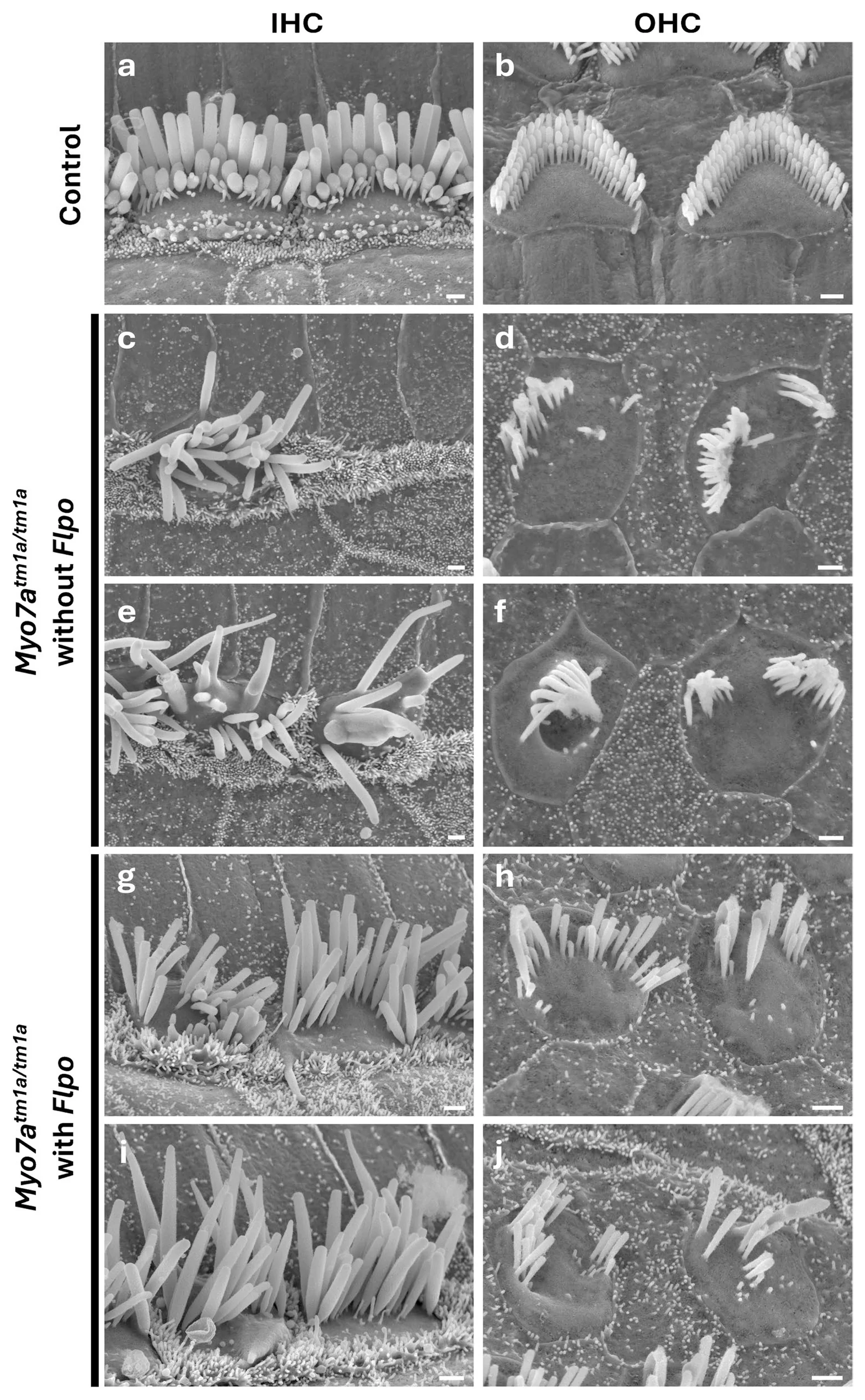
*Myo7a*^*tm1a/tm1a*^ mice have severely disorganised IHC and OHC stereocilia Scanning electron microscopy images of IHCs and OHCs from the 12 kHz organ of Corti frequency region of nine week old mice which had received tamoxifen at P4 via the mother; **a** and **b** control mice (*Myo7a*^+*/tm1a*^ with or without Flpo), **c**-**f** *Myo7a*^*tm1a/tm1a*^ without *Flpo*, **g**-**j** *Myo7a*^*tm1a/tm1a*^ with *Flpo*. All mice received tamoxifen regardless of genotype. Scale bars = 1 µm. *N* = 3 for each genotype

As previously studied *Myo7a* mouse mutants have disorganised stereocilia bundles [11, 26], scanning electron microscopy was used to investigate the stereocilia organisation in the *Myo7a*^*tm1a*^ mutant aged nine weeks old. The hair bundles of IHCs and OHCs from control mice were all formed of 3 rows of stereocilia in the correct staircase organisation (Fig. 5a and b). Consistent with their profound deafness, both *Myo7a*^*tm1a/tm1a*^ mice with and without *Flpo* have severely disorganised stereocilia bundles on IHCs and OHCs at the 12 kHz and 36 kHz best-frequency regions (Fig. 5c-f). This was accompanied by substantial hair cell loss, particularly at 36 kHz where the sensory epithelium was almost entirely smooth (Fig. 5d and f). Higher magnification images at the 12 kHz region revealed stereocilia morphology was also markedly impacted by the *Myo7a*^*tm1a*^ mutation (Fig. 6c-j). Stereocilia on IHCs were often excessively long and fused together in *Myo7a*^*tm1a/tm1a*^ mice (Fig. 6c, e, g and i). Likewise, stereocilia on OHCs were also frequently fused and formed clusters on the cell surface (Fig. 6d, f, h, j). Unsurprisingly, given the lack of Myo7a expression in mice following tamoxifen administration at P4, we did not observe any rescue in stereocilia morphology or organisation of hair cells in *Myo7a*^*tm1a/tm1a*^ with *Flpo* compared to *Myo7a*^*tm1a/tm1a*^ without *Flpo*.

## Discussion

### The *Myo7a*^*tm1a*^ Allele Causes Early Onset Profound Hearing Loss

Here we have shown *Myo7a*^*tm1a*^ is a recessive allele which results in profound deafness at all frequencies tested in mice from four weeks onwards. This is similar to many previously studied *Myo7a* mutants such as *Myo7a*^*6J*^, *Myo7a*^*816SB*^ and *Myo7a*^*tm1b*^ which also cause profound deafness [11, 13]. The *Myo7a*^*tm1b*^ allele is similar in design to *Myo7a*^*tm1a*^ except the floxed critical exons (10 and 11) have been completely removed by exposure to Cre recombinase. Intriguingly, unlike the *Myo7a*^*tm1a*^ mutant studied here, the *Myo7a*^*tm1b*^ mutant is not recessive, with *Myo7a*^+*/tm1b*^ heterozygotes exhibiting a mild high frequency loss at 5 months of age [13]. Similarly, *Myo7a*^+*/sh1*^ mice exhibit raised ABR thresholds at six months old [17]. These differences could be explained by interactions with the mouse mutant genetic background as the *Myo7a*^*tm1a*^ mice used here are on a C57BL/6N background with a repaired *Cdh23*^*ahl*^ allele (*Cdh23*^*ahl*+*em3H*^) while the *Myo7a*^*tm1b*^ mice were tested on an unrepaired *Cdh23*^*ahl*^ background. Nonetheless, our findings suggest that 37% of normal levels of *Myo7a* transcript in *Myo7a*^+*/tm1a*^ heterozygotes is sufficient for normal auditory function, while 17% of normal levels as in *Myo7a*^*tm1a/tm1a*^ homozygotes was not sufficient; presumably there is a threshold between 17 and 37% for the level of *Myo7a* transcript that can support normal hair cell development and function (at least up to six months old, the oldest stage we tested).

As in many other mouse mutants and other animals with hearing loss, there is progression of hair cell loss with age, with outer hair cells being more vulnerable to degeneration than inner hair cells (compare Fig. 2f-q at 4 weeks old with Fig. S2c at 9 weeks old and Fig. 4b, d, e at 15 weeks old) [27, 28]. However, this hair cell degeneration cannot account for the raised ABR thresholds because the mice are deaf but have many intact hair cells at 4 weeks old.

### Activation of Myo7a Expression at Four Weeks Old Results in Some Response to Sound

Here, we have shown that tamoxifen injections at four weeks old activates Myo7a expression in *Myo7a*^*tm1a/tm1a*^ with *Flpo* mice and this produces an ABR at 12 and 18 kHz in mice which previously did not respond to sound stimuli at all. The lack of substantial hearing rescue we observed is consistent with a dual-AAV vector-based attempt to restore expression of wildtype Myo7a which improved cochlear hair cell survival but not auditory function in the *Myo7a*^*4626SB/4626SB*^ mutant [16]. However, two other studies aiming to restore hearing in the *Myo7a*^*sh1/sh1*^ mutant have succeeded in recovering some hearing functionality. The first of these used a lentiviral strategy to deliver full-length cDNA of the wildtype human *MYO7A* canonical isoform to the inner ears of *Myo7a*^*sh1/sh1*^ homozygotes. Similar to our study, this report found improvements in ABR thresholds when mice were treated at P16 but not when they were treated at the younger age of P4 [17]. It may be that the *Myo7a* gene in P4 hair cells is not in a state to respond to such treatments, or the inner ear may be less accessible to the drug. The second study used a dual-AAV approach to deliver the full length of the murine *Myo7a* cDNA at P0-1. The authors observed a reduction in ABR thresholds between 6–30 kHz coupled with a rescue of stereocilia organisation [29].

The differing levels of hearing restoration across these studies and ours is likely due to two main reasons: firstly, the severity of the impact of the *Myo7a* mutation which is used and, secondly, the mouse age at treatment. *Myo7a*^*4626SB*^ is a nonsense mutation in the head domain resulting in a lack of protein expression [30] and severe hair cell defects [31], whereas *Myo7a*^*sh1*^ is an arginine-to-proline missense mutation with normal levels of Myo7a expression [30] and only minor abnormalities in stereocilia organisation up to P10-15 [11, 29]. The *Myo7a*^*tm1a*^ allele used in this study results in no detectable Myo7a protein expression and so can be considered most similar to the *Myo7a*^*4626SB*^ from these gene therapy studies.

Our approach using *Flp* recombinase to activate the *Myo7a* gene has the limitation that it can only be a proof-of-concept as humans do not carry the same mutation as in the *Myo7a*^*tm1a*^ allele. Furthermore, the resulting activation was patchy as not all hair cells showed expression of Myo7a protein, and tamoxifen injection at P4 was unsuccessful in producing any Myo7a protein in the cochlea. However, gene therapy approaches to activating genes also usually show patchy expression of the targeted gene in the cochlea. We think it is likely that the poor thresholds we detected are probably due to the failure to repair the stereocilia bundles rather than our finding that not all hair cells showed Myo7a expression after tamoxifen exposure.

There is a huge range of *MYO7A* mutations in humans responsible for DFNA11, DFNB2, and USH1B [32] so it is important to study a range of mouse mutants to determine potential therapeutic interventions. Myo7a is necessary for correct stereocilia development [11, 26], as well as mechanoelectrical transduction (MET) channel maintenance [33]. Thus, if the stereocilia bundles do not develop correctly in the first place in *Myo7a* mutants, it will be extremely challenging to reverse the subsequent hearing loss, particularly at a clinically relevant age. This has also been shown to be the case with mutations in other hair cell specific genes which are essential for early developmental stages and cochlear maturation [34].

## Supplementary Material

**Supplementary Information** The online version contains supplementary material available at https://doi.org/10.1007/s10162-026-01050-2.

Supplementary data

## Figures and Tables

**Table 1:** Primers used for short-range PCR genotyping

Reaction Name	Forward Primer	Reverse Primer	Product size (bp)	Interpretation
*Myo7a* wildtype	***Myo7a*F—**GGG AGA GAA AGC AGG GTG TG	***Myo7a*R—**AAG CTG GAC TCT CTG GTG GC	360	Wildtype *Myo7a* allele present
*Myo7a* tmla cassette	***Myo7a*F—**GGG AGA GAA AGC AGG GTG TG	**CasRl—**TCG TGG TAT CGT TAT GCG CC	178	tm1a allele present at *Myo7a* locus
*ROSA26* wildtype	***ROSA26*F—**AAA GTC GCT CTG AGT TGT TAT	***ROSA26*R—**GGA GCG GGA GAA ATG GAT ATG	603	Wildtype *ROSA26* locus
*ROSA26 Flpo*	***ROSA26*F—**AAA GTC GCT CTG AGT TGT TAT	***Flpo*R—**TTA TGT AAC GCG GAA CTC CA	309	*FlpoER* gene present at *ROSA26 *locus
*LacZ* presence	***lacZ*F—**ATC ACG ACG CGC TGT ATC	***lacZ*R—**ACA TCG GGC AAA TAA TAT CG	108	*LacZ* gene is present (tm1a cassette is present)

## Data Availability

Not applicable.
