# Supplementary material for: Limited Potential to Reverse Deafness Caused by Mutation of Myo7a
Source: J Assoc Res Otolaryngol. Author manuscript; Available in PMC 2026 May 27. (PMC7619100; doi:10.1007/s10162-026-01050-2)
Supplement: Supplementary data [file EMS213865-supplement-Supplementary_data.pdf]

Limited potential to reverse deafness caused by mutation of *Myo7a*

Daniel R. Pentland, Jack Blackburn, Lauren Witting, Darcey A. Kirwin, Karen P. Steel

Supplementary Figure 1

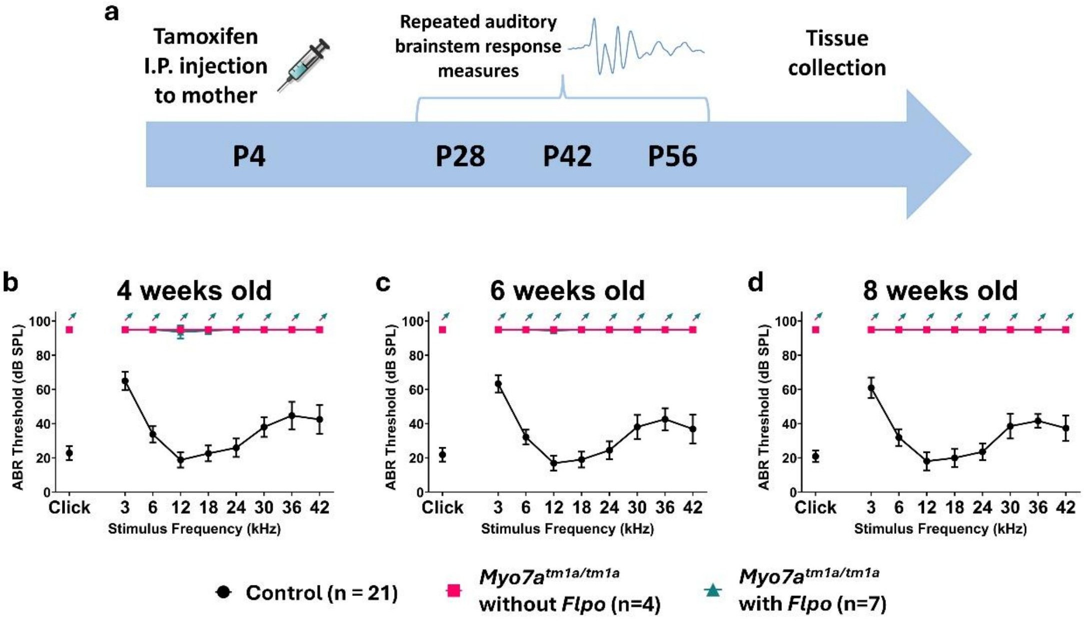

Supplementary Fig. 1 Tamoxifen administration at P4 does not recover hearing loss of *Myo7a*$^{tm1a/tm1a}$ mice (a) Schematic showing the experimental timeline for the P4 cohort; pups received tamoxifen from P4 via their mother's milk, ABRs were performed at four, six and eight weeks old prior to inner ear collection. (B) ABR thresholds of control (black), *Myo7a*$^{tm1a/tm1a}$ without *Flpo* (pink), and *Myo7a*$^{tm1a/tm1a}$ with *Flpo* (teal) mice at four, six and eight weeks old between 3-42kHz. Data points indicate the lowest sound level (mean ± 1 SD) at which features of the ABR waveform start to appear. Data points at 95dB SPL indicate no response up to the highest sound level used. Responses of both *Myo7a*$^{tm1a/tm1a}$ with and without *Flpo* mice were significantly raised compared to control mice ($p < 0.01$, Kruskal-Wallis test). There was no significant difference between responses of *Myo7a*$^{tm1a/tm1a}$ with and without *Flpo* mice.

Supplementary Figure 2

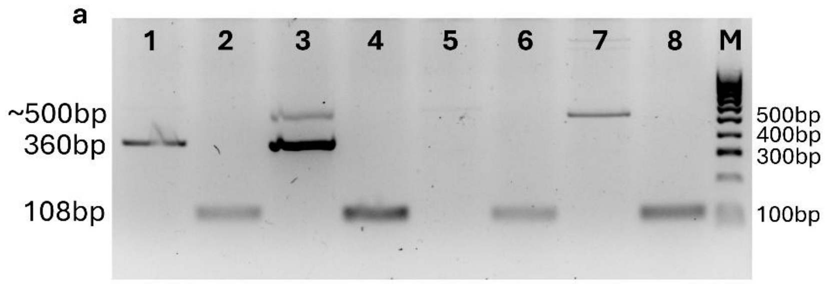

Well	Genotype	*Myo7a*$^{+}$ band (360bp)	*Myo7a*^{tm1c} band (~500bp)	*LacZ* band (108bp)
1	*Myo7a*$^{+/tm1a}$ without *Flpo*	✓	×	-
2		-	-	✓
3	*Myo7a*$^{+/tm1a}$ with *Flpo*	✓	✓	-
4		-	-	✓
5	*Myo7a*$^{tm1a/tm1a}$ without *Flpo*	×	×	-
6		-	-	✓
7	*Myo7a*$^{tm1a/tm1a}$ with *Flpo*	×	✓	-
8		-	-	✓

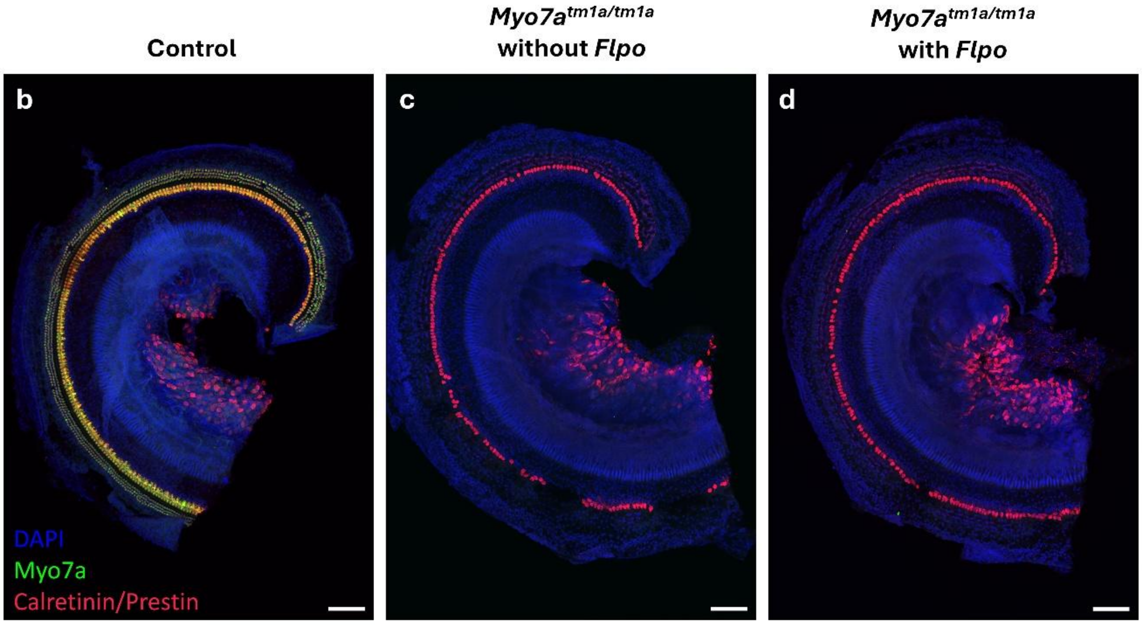

Supplementary Fig. 2 Tamoxifen administration via mother's milk to pups at P4 does not result in Myo7a protein expression in *Myo7a*$^{tm1a/tm1a}$ with *Flpo* mice (a) Agarose gel showing the genomic PCR product using pinna tissue, showing the excision of the tm1a cassette: *Myo7a* F and *Myo7a* R primers produced either a 360bp product from the wildtype *Myo7a*$^{+}$ allele or a ~500bp product from the *Myo7a*^{tm1c} allele if the tm1a cassette has been

excised. Thus, genomic template from *Myo7a*$^{tm1a/tm1a}$ with *Flpo* mice produced a single ~500bp band, and template from *Myo7a*$^{tm1a/tm1a}$ without *Flpo* mice did not produce a band. If a control *Myo7a*$^{+/tm1a}$ mouse carried *Flpo,* 2 bands of 360bp and ~500bp were produced. If any unexcised tm1a cassette remains, the *LacZ* F and *LacZ* R primers produced a 108bp product from the *Myo7a*^{tm1a} allele. (b-d) Confocal images of apical pieces of organ of Corti whole mounts from control, *Myo7a*$^{tm1a/tm1a}$ without *Flpo*, and *Myo7a*$^{tm1a/tm1a}$ with *Flpo* mice at nine weeks old. Tamoxifen was administered to all mice via mother's milk from P4 regardless of genotype. IHCs and OHCs labelled with anti-myoVIIA (both - green), anti-calretinin (IHCs - red), anti-prestin (OHCs – red), nuclei labelled with DAPI (blue). Scale bars = 100μm.
